# Supplementary material for: Smooth Interpolating Curves with Local Control and Monotone Alternating Curvature
Source: Comput Graph Forum. 2022 Oct 6;41(5):25–38. doi: 10.1111/cgf.14600 (PMC9827861; doi:10.1111/cgf.14600)
Supplement: Supplementary file 1 — Supplement Material [file CGF-41-25-s001.zip › Local-Smooth-Interpolating-MonoCurvature/extern/clothoids/docs/api-matlab/program_listing_file_CompileOctave.m.html]

Program Listing for File CompileOctave.m — Clothoids v2.0.9

### Navigation

- index
- toc
- Clothoids »
- Program Listing for File CompileOctave.m

# Program Listing for File CompileOctave.m¶

↰ Return to documentation for file (`CompileOctave.m`)

```
clc;
clear functions;

NAMES = { ...
  'FresnelCS', ...
  'XY_to_angle', ...
  'CircleArcMexWrapper', ...
  'BiarcMexWrapper', ...
  'BiarcListMexWrapper', ...
  'LineSegmentMexWrapper', ...
  'PolyLineMexWrapper', ...
  'Triangle2DMexWrapper', ...
  'ClothoidCurveMexWrapper', ...
  'ClothoidListMexWrapper', ...
  'ClothoidSplineG2MexWrapper', ...
  'TriTriOverlap'
};

LIB_NAMES = { ...
  'G2lib', ...
  'G2lib_intersect', ...
  'AABBtree', ...
  'Line',...
  'PolyLine', ...
  'Circle', ...
  'Biarc', ...
  'BiarcList', ...
  'Clothoid', ...
  'ClothoidList', ...
  'ClothoidDistance', ...
  'ClothoidG2', ...
  'Fresnel', ...
  'Triangle2D', ...
};

LIB_NAMES2 = { ...
  'PolynomialRoots-1-Quadratic', ...
  'PolynomialRoots-2-Cubic', ...
  'PolynomialRoots-Utils', ...
};

if isunix
  MEX = 'mkoctfile --mex -O2';
elseif ispc
  MEX = 'mkoctfile --mex';
end

INC = ' -I../submodules/quarticRootsFlocke/src -I../src ';

LIB_SRCS = '';
LIB_OBJS = '';
for k=1:length(LIB_NAMES)
  LIB_SRCS = [ LIB_SRCS, ' ../src/', LIB_NAMES{k}, '.cc' ];
  LIB_OBJS = [ LIB_OBJS, LIB_NAMES{k}, '.o ' ];
  CMD = [ MEX, ' -c ', INC, '../src/', LIB_NAMES{k}, '.cc' ];
  disp(CMD);
  eval(CMD);
end
for k=1:length(LIB_NAMES2)
  LIB_SRCS = [ LIB_SRCS, ' ../submodules/quarticRootsFlocke/src/', LIB_NAMES2{k}, '.cc' ];
  LIB_OBJS = [ LIB_OBJS, LIB_NAMES2{k}, '.o ' ];
  CMD = [ MEX, ' -c ', INC, '../submodules/quarticRootsFlocke/src/', LIB_NAMES2{k}, '.cc' ];
  disp(CMD);
  eval(CMD);
end

disp('---------------------------------------------------------');

for k=1:length(NAMES)
  N=NAMES{k};
  disp('---------------------------------------------------------');
  fprintf(1,'Compiling: %s\n',N);
  CMD = [ MEX ' -output ../matlab/', N, ' -I../src ../src_mex/mex_', N, '.cc ', LIB_OBJS ];
  if isunix
    CMD = [CMD, ' -lstdc++ -O2'];
  elseif ispc
  end
  disp(CMD);
  eval(CMD);
end

for k=1:length(LIB_NAMES)
  delete([ LIB_NAMES{k}, '.o' ]);
end

disp('Install struct, io, statistics, optim package, it may take long time');

pkg install -forge struct
pkg install -forge io
pkg install -forge statistics
pkg install -forge optim

disp('----------------------- DONE ----------------------------');
```

### Quick search

### Table of Contents

- Matlab Interface Manual
- C++ API
- MATLAB API

«
hide menu

menu
sidebar
»

### Navigation

- index
- toc
- Clothoids »
- Program Listing for File CompileOctave.m

© Copyright 2021, Enrico Bertolazzi and Marco Frego.
Created using Sphinx 4.2.0.
